# Similar Carcass Surface Microbiota Observed Following Primary Processing of Different Pig Batches

**DOI:** 10.3389/fmicb.2022.849883

**Published:** 2022-05-27

**Authors:** Charlotte Braley, Philippe Fravalo, Marie-Lou Gaucher, Guillaume Larivière-Gauthier, Fanie Shedleur-Bourguignon, Jessie Longpré, Alexandre Thibodeau

**Affiliations:** ^1^Chaire de Recherche en Salubrité des Viandes (CRSV), Faculté de Médecine Vétérinaire, Université de Montréal, Saint-Hyacinthe, QC, Canada; ^2^Département de Pathologie et Microbiologie, Faculté de Médecine Vétérinaire, Université de Montréal, Saint-Hyacinthe, QC, Canada; ^3^Groupe de Recherche et d’Enseignement en Salubrité Alimentaire (GRESA), Faculté de Médecine Vétérinaire, Université de Montréal, Saint-Hyacinthe, QC, Canada; ^4^Le Conservatoire National des Arts et Métiers (CNAM), Paris, France; ^5^Center de Recherche en Infectiologie Porcine et Avicole (CRIPA), Faculté de Médecine Vétérinaire, Université de Montréal, Saint-Hyacinthe, QC, Canada; ^6^F. Ménard, Division d’Olymel s.e.c., Ange-Gardien, QC, Canada

**Keywords:** pig slaughterhouse, pork carcass, farm influence, microbiota, primary processing

## Abstract

Bacterial contamination during meat processing is a concern for both food safety and for the shelf life of pork meat products. The gut microbiota of meat-producing animals is one of the most important sources of surface contamination of processed carcasses. This microbiota is recognized to vary between pigs from different farms and could thus be reflected on the bacterial contamination of carcasses at time of processing. In this study, the microbiota of 26 carcasses of pigs originating from different farms (i.e., batches) were compared to determine if an association could be observed between carcass surface microbiota (top and bottom) and the origin of slaughtered animals. The microbiota of the top and bottom carcass surface areas was analyzed by culturing classical indicator microorganisms (mesophilic aerobic bacteria, Enterobacteria, *Escherichia coli, Pseudomonas*, and lactic bacteria), by the detection of *Salmonella*, and by 16S rRNA gene sequencing. Culture results showed higher Enterobacteria, *E. coli*, and lactic bacteria counts for the bottom areas of the carcasses (neck/chest/shoulder) when compared to the top areas. *Salmonella* was not detected in any samples. Globally, 16S rRNA gene sequencing showed a similar composition and diversity between the top and bottom carcass areas. Despite the presence of some genera associated with fecal contamination such as *Terrisporobacter, Escherichia-Shigella, Turicibacter, Clostridium sensustricto1*, and *Streptococcus* on the carcass surface, sequencing analysis suggested that there was no difference between the different batches of samples from the top and bottom areas of the carcasses. The primary processing therefore appears to cause a uniformization of the carcass global surface microbiota, with some specific bacteria being different depending on the carcass area sampled.

## Introduction

The production of proteins derived from animal sources is an important part of the food chain for humans. Pork products are a growing part of the world economy. Since 1961, the world pork production has increased by four to five times, reaching 112 million tons in 2014 (Ritchie and Roser, 2017). Moreover, consumers are demanding premium food commodities, forcing producers to further reduce bacterial contamination on pig carcasses to improve the quality of the final pork meat products. Bacteria from different sources such as the skin and the digestive tract of the animals, the slaughterhouse environment and equipment contribute to the constitution of the carcass microbiota (Biasino et al., 2018; Zwirzitz et al., 2020) during processing. In pig meat processing, evisceration is universally recognized as a critical step that often results in carcass contamination by bacterial populations, particularly when the application of good practices is either lacking or sub-optimal (Rivas et al., 2000). Therefore, the gut microbial community is thought to be the most important source of carcass contamination by both non-pathogenic and pathogenic bacteria during the pigs meat processing in commercial conditions (Wheatley et al., 2014). The presence of these microorganisms can speed-up meat spoilage, a process that ultimately leads to products that are not suitable for human consumption, in turn leading to economic losses and food waste (Gram et al., 2002). This also impacts the microbial safety of pork meat products, raising concerns over associated foodborne diseases (Borch et al., 1996).

*Salmonella* is one of the most common foodborne pathogens, and contaminated pork meat products are a major source of human foodborne infections, especially in Europe and in the United States (US) (Martínez-Avilés et al., 2019). For example, in the United States, the Centers for Disease Control and Prevention (CDC) estimated that *Salmonella* caused 35% of the illnesses linked to pork meat (Self et al., 2017). In European countries, the prevalence of *Salmonella* on pig carcass surfaces ranged from 0.35% to 17.41% (Pala et al., 2019). The most effective interventions that are recognized to contribute to lowering the presence of *Salmonella* on pig carcasses at the slaughterhouse plant level are the rigorous application of good hygiene practices as well as the slaughter of *Salmonella*-free animals [Bonardi, 2017; Regulation (EC) No 2073/2005, 2005]. Indeed, *Salmonella* is often found within the pig intestinal microbiota without causing any clinical signs in the colonized animals.

The presence of foodborne pathogens on carcasses is routinely accompanied by spoilage microorganisms. Spoilage is defined as unfavorable changes of the organoleptic properties of the meat—such as off-flavors, texture, and poor taste (Casaburi et al., 2015)—rendering the meat unsuitable for human consumption. The initial number and type of microorganisms directly influence the time needed to reach a sufficient level to cause these changes (Gram et al., 2002; Wheatley et al., 2014). Psychrotrophic aerobes and facultative anaerobes such as members of the Enterobacteria, *Pseudomonas spp.*, and lactic acid bacteria (Pothakos et al., 2015) are examples of spoilage microorganisms that originate from the food processing environment and the intestinal microbiota of the animals (Wheatley et al., 2014).

The swine gut microbiota is dominated by Firmicutes, Bacteroidetes, and Proteobacteria at the phylum level (Wang et al., 2019). This gut microbiota plays important roles in the animal host metabolism, immune system development, and resistance to pathogens (Fouhse et al., 2016). Several factors can influence the composition of the gut microbiota: genetics, diet, age of the host, antibiotic treatments, and the presence of foodborne pathogens such as *Salmonella* (Hasan and Yang, 2019). The gut microbiota has also been shown to vary between pigs originating from different farms (Yang et al., 2018).

Until now, most studies conducted on carcass microbiological quality at slaughterhouses focused on the transmission of gut bacteria to the pig carcass surface using culture-dependent methods such as the counting of fecal indicator bacteria that indicate the microbial quality of the final meat product. Studies showed that these fecal indicators were not uniformly distributed on the pig carcass surface, that the level of contamination was higher on the bottom (neck) area, and that this contamination varied between carcasses (Wheatley et al., 2014; Biasino et al., 2018). However, results generated using these culture-based approaches allow for the study of a small fraction of the bacterial communities present. The introduction of high-throughput sequencing technologies has profoundly contributed to the advancement of knowledge in this field, permitting the simultaneous detection of hundreds of bacterial genera for which culturing is not always suitable. A limited number of studies describing the microbiota of pig carcasses using high-throughput sequencing technologies have already revealed the usefulness of this approach and have shed new light on the diversity of the bacterial populations present on pig carcasses. For example, a study by Jakobsen et al. (2019) described the bacterial community transfer from pig tonsils to carcass surface and identified specific bacterial groups that were indicative of a bacterial transfer between these two anatomical regions. Another study by Peruzy et al. (2021) showed that various areas (ham, belly, back and jowl) of the surface of different pig carcasses were dominated by the same bacterial communities, though this microbiota varied between slaughterhouses.

To the best of our knowledge, no study has looked at the influence of the animal origin on the carcass surface microbiota following primary processing of pigs slaughtered in commercial conditions on the same day. Indeed, intestinal microbiota greatly varies between animals originating from different farms (Yang et al., 2018), and on-farm intestinal microbiota manipulation is being applied to increase animal health and lower the incidence of foodborne pathogens. It is therefore important to assess if the farm origin impacts carcass microbiota to gain insight into whether different on-farm interventions might also affect the microbiota of pork products. Therefore, the main objective of this study was to observe if pig carcass surface microbiota could be associated with the origin (batch) of animals. A comparison of carcass surface microbiota between samples of top and bottom areas from different batches was therefore carried out using both culture-dependent and high-throughput sequencing.

## Materials and Methods

### Slaughterhouse

Carcass surface samples were collected at a pig slaughterhouse in Québec, Canada. Before slaughtering, pigs were kept in separate lairage pens for 3 h. The animals were rendered unconscious by carbon dioxide stunning. After bleeding, the carcasses were scalded for 7 min in hot water (temperature varied between 59.5°C and 64°C). Carcasses were then scraped, dehaired, and buckled before being pre-washed with clean water and inspected by a veterinarian. Carcasses were then eviscerated and washed again before cooling (24 h). The processing time between stunning and the start of cooling was 32 min. The number of pigs slaughtered per hour was 649.

### Sampling and Sample Preparation

A total of 26 pig carcasses from 6 different batches were sampled at the end of carcass dressing, just before the final wash that precedes cooling. All steps until this final wash are considered in this study as primary processing. Throughout the manuscript, the term batch refers to animals raised in the same infrastructure (farm) at the same geographical address, of the same age and slaughtered at the same time. Being raised under a same integrated company, pigs from each farm were similar in terms of genetics and productivity and had been fed a similar diet. Batches were numbered 1–6, though pigs sampled in this study were not slaughtered in this order. For each batch, 4 carcasses were sampled, except for the first 2 batches where 5 carcasses were sampled. All samples were collected on the same day over 5 consecutive hours of production. During primary processing, pigs were hung by the back feet. For each carcass, one sample was collected from the top (thighs/buttock) and one sample from the bottom (neck/chest/shoulder). A total of 26 top carcass surface areas and 26 bottom carcass surface areas were sampled. Sterile wipes were first humidified with 10 mL of sterile PBS (ThermoFisher scientific, Ottawa, Canada) prior to sampling and put in a sterile bag. For each sample, an approximate 600 cm^2^ area was firmly swabbed 10 times horizontally and 10 times vertically. Between each area samples, handler’s glove were changed. Three negative controls (wipes exposed to the atmosphere of the slaughterhouse without touching any surfaces) and three external slaughterhouse controls (wipes humidified with 10 ml of sterile PBS and used to swab the floor of the slaughterhouse) were also included during sampling. All samples were kept on ice until processing at the laboratory. Wipes were homogenized in 50 mL of cold buffer (1 mM EDTA, 10 mM Tris–Hcl, 8.5 g NaCl pH 8), homogenized using a stomacher Smasher™ AESAP1064 (Bioméreux, United States) for 1 min and kept on ice. From this volume, 5 mL was immediately used for bacterial enumeration while 30 mL was centrifuged for 20 min at 2,800 *g* (Sorvall legend X1R centrifuge, ThermoFisher scientific, United States). The supernatant was discarded and the dry pellet stored at −80°C until DNA extraction.

### Bacterial Enumeration and Confirmation

For each microbial indicator, 100 μl from each sample were directly plated using a spiral seeder (Spiral Interscience, ThermoFisher scientific, United States) on the appropriate culture medium (Table 1).

**TABLE 1:** Culture conditions for the enumeration of mesophilic aerobic bacteria, Enterobacteria, *Escherichia coli*, lactic bacteria, and *Pseudomonas.*

Microorganisms	Culture media	Culture conditions	According to the procedure derived from
Mesophilic aerobic bacteria	Trypticase Soy Agar	30°C, 48 h	ISO 4833-2:2013
	(BD Difco, Franklin Lakes, NJ, United States)		
Enterobacteria	Violet Red Bile Glucose Agar	37°C, 48 h	ISO 21528-2:2017
	(BD Difco)		
*Escherichia coli*	MacConkey Agar	37°C, 48 h	(MAPAQ, 2019)
	(BD Difco)		
Lactic bacteria	Man, Rogosa, Sharpe medium	37°C, 48 h	(, 2019)
	(BD Difco)	(GazpakAnaeroGen Thermo Scientific™ Oxoid R)	
*Pseudomonas*	Cephalosporin-Fucidine-Cetrimide	25°C, 48 h	ISO 13720:2010
	(Biokar diagnostic)		

Presumptive *Pseudomonas* colonies were confirmed by a positive oxidase test (Gordon-McLeod Reagent, Sigma-Aldrich). In parallel, control strains of each microbial indicator such as *Escherichia coli ATCC 25922, Pseudomonas aeruginosa ATCC 27853*, and *Lactobacillus salivarius ATCC 1174* were used to validate the method and culture conditions used.

### Detection of *Salmonella*

The detection of *Salmonella* was based on a previous study conducted by our group (Larivière-Gauthier et al., 2019, ISO 6579-1:2017). Briefly, 1 mL of samples were pre-enriched in buffered peptone water (1:10 w:v, 24 h, 37°C). Three drops of 100 μL were selectively enriched on Modified Semi-Solid Rappaport-Vassiliadis Agar (MSRV) (Biokar diagnostic, Beauvais, France) (48 h, 42°C). Two selective media—Brilliant Green Sulfa (BGS) (BD Difco, Franklin Lakes, NJ, United States) and Xylose-Lysine-Desoxycholate (XLD) (Biokar diagnostic)—were inoculated from the migration front of each positive MSRV and incubated for 24 h at 37°C. When possible, two typical colonies from each culture medium were confirmed using triple sugar iron agar slants (BD Difco, Franklin Lakes, NJ, United States), urea agar slants, and seroagglutination with *Salmonella* O antiserum Poly A-I C Vi performed on colonies previously purified on blood agar (Statens Serum Institute, Denmark).

### DNA Extraction

Total DNA was extracted from the pellets and kept at −80°C using a mechanical and chemical lysis followed by phenol/chloroform purification. For each sample, 500 mL of lysis buffer [500 mM Tris–HCl pH 8, 2 mM EDTA pH 8, 100 mM NaCl, and 1% SDS (w/v)] containing 1 g of 0.1 mm glass beads was added to each sample. Cells were mechanically lysed using FastPrep-24™ (MP Biomedicals, Santa Ana, CA, United States) for 40 s at 6 m/s and kept on ice. Lysates were centrifuged for 15 min at 18,000 × *g* (Compact Micro Centrifuges, VWR International, United States) to remove beads and cell debris. For each sample, 300 μL of supernatant was mixed by inversion for 5 min with 300 μL of phenol/chloroform/isoamyl alcohol (25:24:1). After centrifugation (18,000 × *g*, 5 min) (VWR International), the aqueous phase was kept and added to 500 μL of chloroform/isoamyl alcohol (24:1). After centrifugation (18,000 × *g*, 10 min), 350 μL of supernatant was added to 117 μL ammonium acetate (0.1 g/mL) and 934 μL of 90% ethanol. DNA was precipitated overnight at −20°C. After centrifugation (18,000 × *g*, for 15 min), 250 μL of 70% ethanol was added to the pellet, samples were centrifuged, and the supernatant was removed. The DNA pellet was air dried for 30 min and 40 μL of dissolution solution (1 mM Tris–HCl pH 8, 0.1 mM EDTA pH 8) was added. Purified DNA samples were stored at −80°C. After extraction, the final DNA concentration was measured using the Qubit 3.0 broad range assay (Fisher Scientific, Ottawa, ON, Canada) on a Denovix (Wilmington, DE, United States) fluorometer. The purity of DNA was assessed using a Nanodrop (ThermoFisher Scientific, United States). In addition, a negative control was included during DNA extraction (water instead of a carcass swab sample) to assess potential cross-contamination during the extraction step.

### 16S rRNA Sequencing

A 291 pb fragment of the V4 region of the 16S rRNA gene was amplified by PCR using the V4-reverse 5′ACACTGACGAACTGGTTCTACAAGTGCAGCMGCCGCG GTAA 3′ and V4-forward 5′TACGGTAGCAGAGACTTGG TCTGGACTACHUGGGTWTCTAAT 3′ (Caporaso et al., 2012) primers. The PCR reaction mix (30 μl) contained 5X SuperfiBuffer, 5X SuperfiGCenhancer, 2 U/μL Platinum Superfi DNA Polymerase (Invitrogen, Burlington, ON, Canada), 10 mM dNTPmix (Bio Basic Inc., Markham, ON, Canada), 20 μM of primer (Invitrogen), 20 mg/mL BSA (ThermoFisher scientific, Ottawa, Canada), 12.5 ng of DNA, and sterile water to reach final volume. The amplification was carried out for 25 cycles and included a denaturation step at 95°C for 30 s, an annealing step at 55°C for 30 s, and an elongation step at 72°C for 60 s in a Mastercycler^®^ Nexus (Eppendorf AG, Hamburg, Germany). These cycles were preceded by an initial denaturation of 5 min at 95°C and followed by a final elongation of 10 min at 72°C. A positive control containing DNA from eight known bacterial DNA with different 16S rRNA gene abundance (theorical composition based on 16S sequencing: 18.4% *Lactobacillus*, 17.4% *Bacillus*, 15.5% *Staphylococcus*, 14.1% *Listeria*, 10.4% *Salmonella*, 10.1% *Escherichia*, 9.9% *Enterococcus*, and 4.2% *Pseudomonas*) was included (ZymoBIOMICS Microbial Community DNA Standard, Zymo Research, Irvine, CA, United States). A PCR negative control (water instead of DNA) was included to assess potential cross-contamination during the PCR step. PCR amplification was confirmed by migration on a 1.5% agarose gel. Amplicons were sent for sequencing (Illumina MiSeq, PE 250) to McGill University and Genome Québec Innovation Center, Montréal, Canada.

### Sequencing Data Processing and Analysis

Raw sequence reads were cleaned and analyzed using Mothur (Schloss et al., 2009) version 1.43 following the MiSeq standard operational procedure^1^ with some modifications, as described in Kozich et al. (2013). The Deblur algorithm was used to complete preclustering. Sequences were aligned against SILVA 132 reference database^2^. Chimeras were removed using VSEARCH (Rognes et al., 2016). The resulting sequences were classified against the SILVA 132 reference database (formatted for Mothur). Sequences were clustered into Operational Taxonomic Units with a unique method, therefore Amplicon Sequence Variants (ASV) were used for analysis. Taxonomic assignation was performed with the Ribosomal Database Project (RDP) trainset 16 database^3^. Alpha diversity indexes (Observed ASV, Shannon and Inverse Simpson) were calculated using the R package “phyloseq” (McMurdie and Holmes, 2013). Beta diversity was analyzed using Jaccard and Bray-Curtis dissimilarity indexes, and the microbiota structure was visualized using non-metric multidimensional scaling (NMDS) graphs in RStudio (version 1.4.1103).

### Statistical Analysis

Total bacterial counts were converted into log_10_ CFU/600 cm^2^ values. Statistical analysis was performed using Rstudio (version 1.4.1103). A *t*-test was used for the comparison of the mesophilic aerobic bacteria mean count between the top and bottom areas of the sampled carcass surfaces. An ANOVA test was conducted for the comparison of mesophilic aerobic counts between the six different batches for samples recovered from both the top and bottom of the carcasses sampled. Graphs were generated with GraphPad Prism 8.0.2. Since counts for some indicator microorganisms were below the detection limit for most samples, the number of positive carcasses for Enterobacteria, *Escherichia coli*, and lactic bacteria between the different batches was analyzed by Fisher exact test. *Salmonella* prevalence was also analyzed by Fisher exact test. A *p* < 0.05 value was considered statistically significant.

Statistical tests related to sequencing data were performed in Rstudio according to our in-house Standard Operating Procedure (SOP) (Larivière-Gauthier et al., 2017). Data were first normalized according to the number of total counts in each sample using the lowest number of sequences found in a carcass sample. Alpha and beta diversity analyses were performed. A *t*-test was used to compare the alpha diversity measures identified from the top and bottom of the sampled carcasses. An Kruskal–Wallis test was performed to compare the alpha diversity measures from all top and bottom surfaces between the six batches sampled.

PERMANOVA tests were conducted using the ADONIS function in the vegan package (Oksanen et al., 2018) for the analysis of the microbiota structure to compare top and bottom carcass surfaces and to assess differences for each type of samples according to batch. Species abundance was compared between groups using Multivariate Association with Linear Models 2 (MaAsLin2) (Mallick et al., 2021) to identify biomarkers associated with sample type (top or bottom) or batches at the phylum, family, and genus levels [using the tax_glom function in the phyloseq package (McMurdie and Holmes, 2013)] after grouping of the 6 batches without rarefaction of the sequences. All analysis in MaAslin2 were performed using default options and an association was considered significant at a *p*-value < 0.05 and *q*-value < 0.25.

## Results

### Enumeration of Bacterial Populations

**FIGURE 1:**
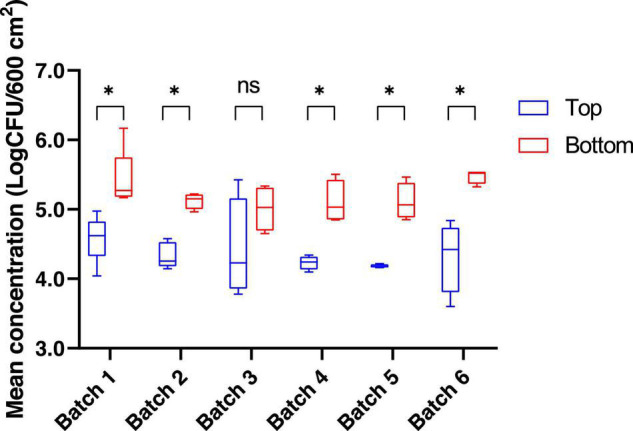
Mean concentration (log CFU/600 cm^2^) for mesophilic aerobic bacteria counts for the top and the bottom areas of the pig carcass surface. *p < 0.05. ns, non-significant.

The bacterial communities present on pig carcass surfaces were investigated for 26 carcasses from 6 different batches. Both the top (thighs/buttock) and bottom (neck/chest/shoulder) areas of the pig carcass were sampled. The mean mesophilic aerobic bacteria concentration for the top and bottom areas of the carcass, regardless of the batch was 4.6 log_10_ CFU/600 cm^2^ and 5.3 log_10_ CFU/600 cm^2^, respectively. When comparing these same mesophilic aerobic bacteria counts obtained from the sampling of the bottom area to those of the top part of the carcass for each batch separately, a significant difference between top and bottom areas was found for 5 out of the 6 batches sampled (*p* < 0.05) (Figure 1). When conducting this comparison at the batch level, no statistically significant difference could be observed for the mesophilic aerobic bacteria counts for both the top and bottom carcass surface areas among the six batches sampled (*p* > 0.05).

Bacterial counts for Enterobacteria, *Escherichia coli*, lactic acid bacteria, and *Pseudomonas* are presented in Table 2. For all of these bacterial populations, the percentage of positive carcasses sampled recovered from the bottom area was significantly higher than the proportion of positive samples identified for the top area of the carcass (*p* < 0.05) (Table 2).

**TABLE 2:** Percentage of positive carcasses and mean bacterial concentrations for Enterobacteria *Escherichia coli*, lactic acid bacteria, *Pseudomonas*, and *Salmonella* for the top and bottom areas of the carcass surface samples analyzed.

	Positive samples (%)		Mean bacterial concentration (log_10_ CFU/600 cm^2^ ± σ)	
	Top	Bottom	Top	Bottom
Enterobacteria	3.8^a^	38.5^b^	1.6 ± 0.2	2.4 ± 1.3
*Escherichia coli*	7.7^a^	34.6^b^	1.6 ± 0.4	2.5 ± 1.2
Lactic acid bacteria	58.0^a^	96.1^b^	3.4 ± 1.5	3.5 ± 0.3
*Pseudomonas*	0	0	<2.7	<2.7
*Salmonella*	0	0	<2.7	<2.7

*Values with different superscripts in a row are significantly different (p < 0.05). For Pseudomonas and Salmonella, the mean bacterial concentrations (log10 CFU/600 cm^2^) were below the detection threshold for the method used. σ, standard deviation. A sample is considered positive if a bacterial count could been performed.*

### Sequence Quality

The V4 region of the 16S rRNA gene was sequenced from sixty-one samples (26 bottom carcasses, 26 top carcasses, 2 negatives controls (extraction and PCR), 3 negative wipe controls, 3 external slaughterhouse controls, and 1 DNA community). After reads processing, a total of 1,449,466 sequences were retained and assigned to 27 phyla, 58 classes, 106 orders, 225 families, and 663 genera. The average number of sequences per sample was 79,421. The total number of ASV was 22,920. The mock community corresponding to the positive control was composed of 17.5% *Salmonella*, 15.9% *Escherichia/Shigella*, 14.6% *Bacillus*, 12.6% *Staphylococcus*, 12.3% *Lactobacillus*, 11.4% *Listeria*, 8.2% *Pseudomonas*, and 6.4% *Enterococcus*. The negative controls used for the DNA extraction and PCR steps contained 798 and 29,980 sequences respectively, and no band was visible on gel electrophoresis.

### Carcass Surface Microbiota Description of the Top and Bottom Areas of Pig Carcasses

**FIGURE 2:**
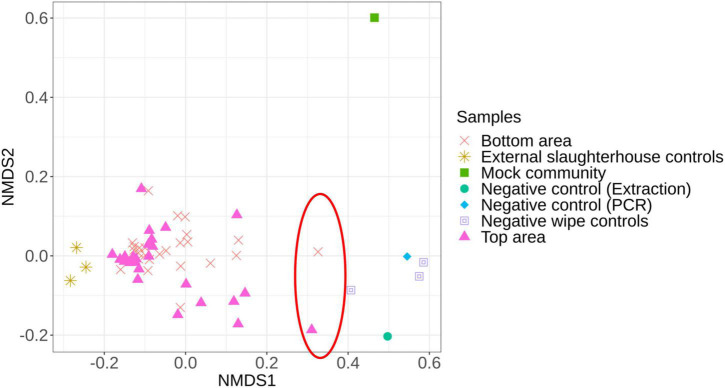
Non-metric multidimensional scaling (NMDS) plot illustrating the microbiota structure between all samples. The red circle identifies the samples removed from the analysis due to their proximity to the negative controls.

First, we compared the microbiota structure between the carcass samples (top or bottom areas), the mock community, the negative controls, and the external slaughterhouse controls to investigate dissimilarities between each sample type (Figure 2). Two samples (1 top sample and 1 bottom sample from different batches) were significantly different from the overall carcass samples collected and relatively close to the negative control samples (Figure 2). These two samples were therefore removed from the subsequent analysis.

**FIGURE 3:**
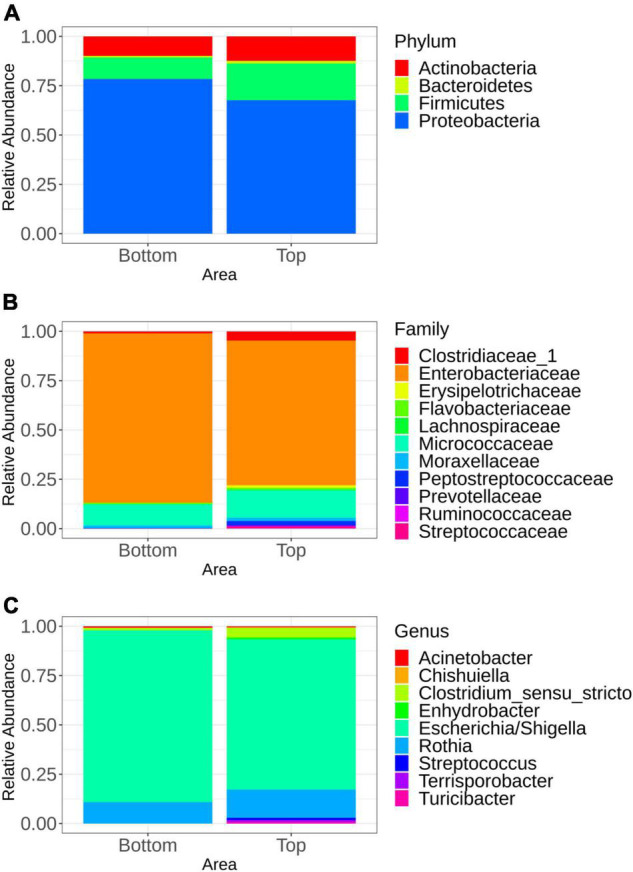
Mean relative abundance of the major bacterial groups at the phylum level **(A)**, family level **(B)**, and genus level **(C)** identified in samples representing the top and bottom areas of the pig carcasses. Only bacterial communities representing at least 5% of carcass surface microbiota are shown.

The microbiota general composition at the phylum, family, and genus levels is illustrated on Figure 3. Overall, regardless of the batch, the major phyla, families, and genus were the same for samples recovered from both the top and bottom areas (Figures 3B,C). In total, 663 genera were assigned from all the sequences analyzed, but only 9 genus had a relative abundance greater than 5% (Figure 3C).

Multivariate association using linear models (MaAslin2) was performed in order to determine which microbial taxa were preferentially associated with the bottom or the top carcass surface, regardless of the batch. Indeed, any microbial taxa were associated with one of the six batches sampled. Samples recovered from the top carcass areas were positively associated with Proteobacteria. Firmicutes were positively associated with samples collected from the bottom area. At the family level, the microbiota of the top area of the carcass was significantly associated with *Bradyrhizobiaceae, Caulobacteraceae*, and *Planctomycetaceae*, while families of *Halomonadaceae, Corynebacteriaceae, Pasteurellaceae, and Aerococcaceae* were found to be significantly associated with its bottom counterpart. At the genus level, *Phenylobacterium* and *Bradyrhizobium* were found to be significantly associated with the top area of the carcass. The presence of *Halomonas, Lactococcus, Aerococcus*, and *Corynebacterium* was significantly associated with samples collected from the bottom area. All significant results (*p* < 0.05) are available in the Supplementary Table 1.

### Microbiota Diversity of the Pig Carcass Surface

**FIGURE 4:**
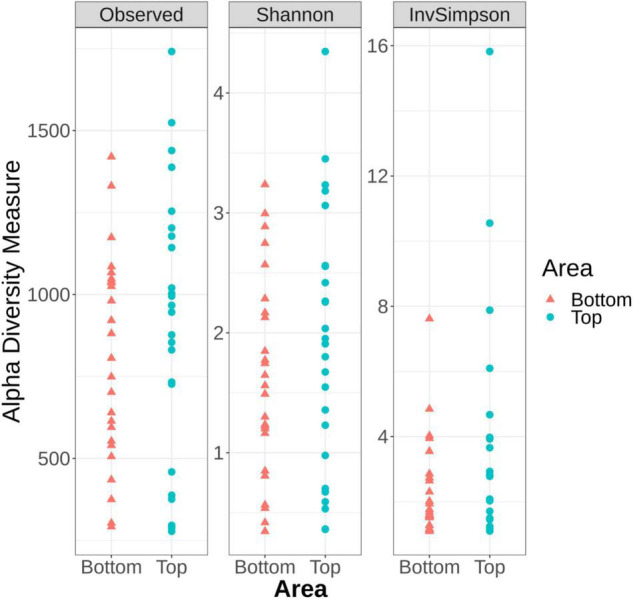
Alpha diversity measures among the top and the bottom areas of the pig carcasses sampled using Observed, Shannon, and Inverse Simpson indices.

Alpha diversity analysis, which describes the bacterial richness and distribution within a sample, was used to compare the surface microbiota between the top and bottom areas of the pig carcasses sampled. Plots representing the alpha diversity measures obtained are shown in Figure 4. No significant differences were observed (*t*-test *p* > 0.05) between the 26 top surface microbiota and the 26 surface microbiota bottom areas samples.

The alpha diversity measures from all top or bottom areas were compared between the six batches sampled and no significant differences between batches could be observed (Kruskal–Wallis test *p* > 0.05) (Supplementary Figures 1, 2). These measures were also compared within each carcass of a same batch. Only the samples collected from the fourth batch sampled showed Observed number of ASVs (sequences of bacteria) and Inverse Simpson indices significantly higher for the top area of the carcass surface when compared to their bottom counterpart (Supplementary Figure 2).

**FIGURE 5:**
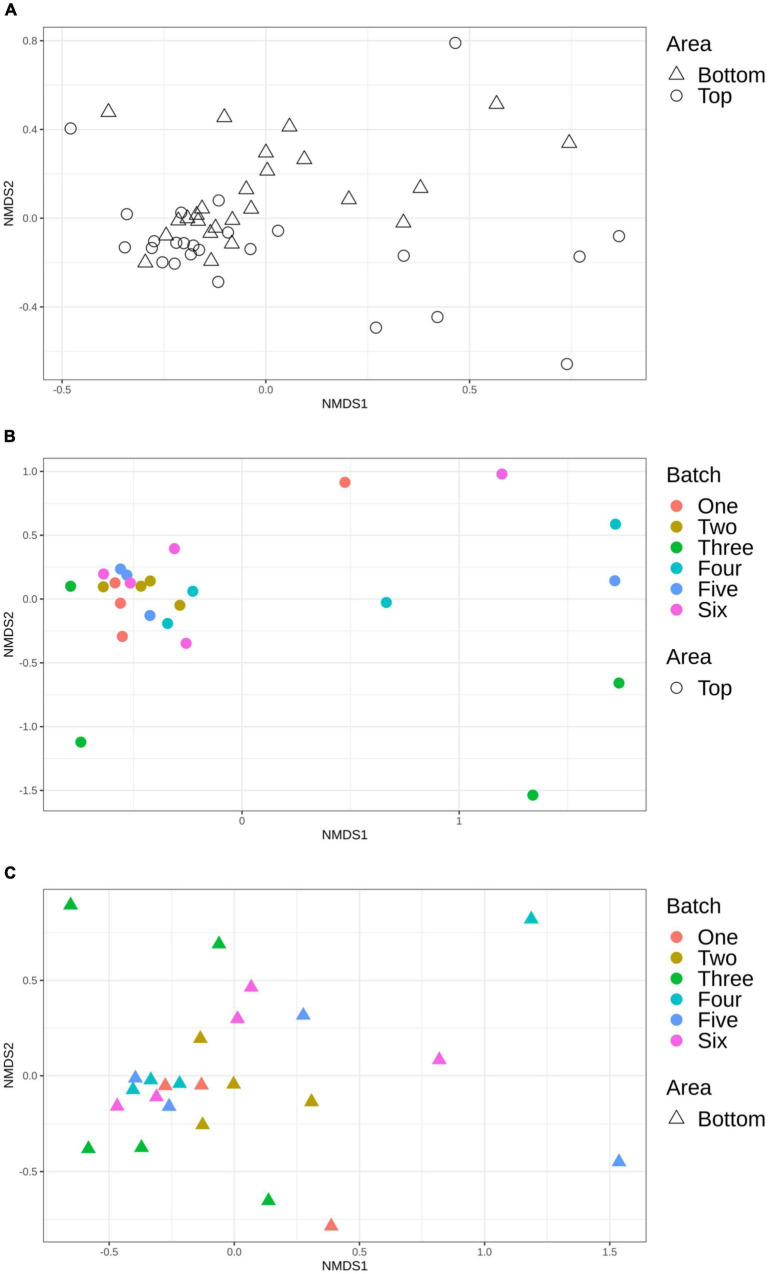
Non-metric multidimensional scaling (NMDS) plot illustrating the microbiome structure of the pig carcass surface according to area **(A)**, top area according to batch **(B)**, and bottom area according to batch **(C)**.

Using Bray-Curtis and Jaccard distance matrices, results showed that the structure and membership of the bacterial community was similar (PERMANOVA *p* > 0.05) between the top and bottom areas of the pig carcasses sampled (Figure 5A). This same observation was made for the comparison of the top or bottom areas among the different batches sampled (Figures 5B,C).

## Discussion

In this study, the microbiota of 26 pig carcass surfaces were analyzed by culture-dependent methods and high-throughput sequencing in order to describe the carcass surface microbiota composition and to determine if an association could be observed between the carcass surface microbiota (top and bottom) and the animal’s origin (batch).

Mesophilic total bacteria, usually regarded as an indicator of the hygiene conditions of the entire meat production process, showed counts that were similar to those reported in previous studies, with values ranging from 3 to 6.4 log CFU/cm^2^ (Martínez et al., 2010; Bohaychuk et al., 2011; Piras et al., 2014). In our study, significant differences between counts for the top and bottom areas of the pig carcasses were identified on the same day of slaughter in the slaughterhouse, regardless of the batch. Similar findings were reported in the literature but the samples were collected over the course of several visits (Spescha et al., 2006; Biasino et al., 2018). The impact of animal batch has been reported for pathogenic bacteria like *Salmonella* and *Yersinia enterocolitica* (Gonzales-Barron et al., 2013; Vanantwerpen et al., 2015) but, to the best of our knowledge, this is the first report for mesophilic total bacteria.

Our results showed similar levels of Enterobacteria and *E. coli*—usually used to assess fecal contamination—to those reported by other studies, with mean levels of 2 log CFU/cm^2^. In several other studies, the bottom parts of the carcasses were reported to be more contaminated than the top after evisceration (Spescha et al., 2006; Zweifel et al., 2008; Martínez et al., 2010; Wheatley et al., 2014).

Lactic acid bacteria and *Pseudomonas* are psychotropic bacteria responsible for meat spoilage (Salifou et al., 2013). A recent study that characterized the pig carcass surface microbiota according to different areas (Peruzy et al., 2021) observed levels of lactic acid bacteria similar to those observed in the present study, around 3.61 log CFU/cm^2^. *Pseudomonas* was not detected in any of our samples and the probable growth of this psychrotrophic bacteria had perhaps not started before the cooling process. Indeed, in many studies, *Pseudomonas spp*. was mainly found on refrigerated pork products after cooling (EFSA Panel on Biological Hazards [BIOHAZ], 2016; Stellato et al., 2016).

In the present study, none of the carcasses sampled was contaminated by *Salmonella*, preventing us from making any association between the presence of the pathogen and the microbiota composition. Other studies are reporting different prevalence levels for *Salmonella* according to meat processing. For example, Piras et al. (2014) and Biasino et al. (2018) reported 64% and 18% of *Salmonella*-positive carcasses after evisceration and immediately after cooling respectively. The application of good slaughtering practices combined with the absence of *Salmonella* in the intestines of pigs slaughtered on the sampling day could explain this negative *Salmonella* status.

Microbiota analysis using high-throughput sequencing revealed similar bacterial communities between the top and bottom areas of the pig carcasses at the phylum level, while comparison at family and genus level showed significant differences in the relative abundance between these two areas. No information pertaining to the difference of phylum and family relative abundances between the top and bottom areas of pork carcasses seems to be available in the scientific literature. However, according to Peruzy et al. (2021), the bacterial community on four carcass areas (jowl, belly, back, ham) was dominated by the same bacterial genera that were also observed in the current study, i.e., *Escherichia* and *Rothia*. These similarities are not surprising as these genera reside in the oral and intestinal microbiota of pigs as well as in slaughterhouse environments.

In our study, *Terrisporobacter, Escherichia-Shigella, Turicibacter, Clostridium sensu stricto*, and *Streptococcus* represented the most abundant bacterial populations found on carcass surfaces, representing more than 90% of the microbiota analyzed. It has been reported that these bacterial populations were present in the gut microbiota of pigs (Quan et al., 2018). Indeed, according to this study by Quan et al. (2018), *Escherichia-Shigella* (23.1%), *Terrisporobacter* (17.9%), and *Clostridium sensustricto1* (12.9%) were most prevalent in the pig ileum, and *Streptococcus* (8.0%) was one of the most prevalent genera in the colon of this animal species. *Turicibacter* was most present in the jejunum and ileum (Crespo-Piazuelo et al., 2018). In our study, *Terrisporobacter, Streptococcus*, and *Turicibacter* were only found on the top area of the sampled carcasses. It is worth noting that carcasses were sampled before washing and cooling in the current study. This could probably explain why fecal contaminants were found on the top surface area since washing usually involves water running down from the top to the bottom part of the carcass, creating a higher risk for contamination in this area (Bolton et al., 2002). Furthermore, several families (*Bradyrhizobiaceae, Caulobacteraceae, Planctomycetace, Halomonadaceae, Corynebacteriaceae, Pasteurellaceae, Aerococcaceae*) and genus (*Phenylobacterium, Bradyrhizobium, Halomonas, Lactococcus, Aerococcus, Corynebacterium*) have been observed and each of them were statistically associated with the top or the bottom carcass surface microbiota. These bacterial populations were all previously reported as members of the pig gut microbiota (Fan et al., 2017; Zwirzitz et al., 2019). The description of bacteria isolated from specific carcass areas allowed for a better understanding of the dispersion of bacteria on the carcass surface as well as for the identification of contamination origin.

The *Flavobacterium* family which is responsible for the occurrence of rancid odors causing meat spoilage—which has been suggested to originate from worker gloves during evisceration—was also present on the surface of pig carcasses sampled in the current study (Doulgeraki et al., 2012; Zwirzitz et al., 2020; Wu et al., 2021).

It is well known that the major source of contamination of the carcass surface is evisceration (Biasino et al., 2018), but the removal of tonsils, tongue, and gallbladder are other important contaminating steps during primary processing (Jakobsen et al., 2019). In the current study, the analysis of the carcass surface microbiota revealed the presence of bacteria such as *Acinetobacter, Enhydrobacter*, and *Rothia. Acinetobacter* was naturally found in the tonsils of pigs (Jakobsen et al., 2019) and was associated with meat spoilage (Iulietto et al., 2015). According to Bridier et al. (2019), *Acinetobacter* was also found in the slaughterhouse environment, especially at the dehairing step, and *Enhydrobacter* was present in the neck clipper environment. In another study, *Acinetobacter* and *Rothia* were observed to be the most dominant genera of the carcass surface microbiota (Peruzy et al., 2021; Wu et al., 2021) and were both common inhabitants of the oral microbiota of pigs.

Finally, in our study, the richness and the structure of carcass surface microbiota appeared similar between the top and bottom areas and between the six different batches sampled. According to the study by Peruzy et al. (2021), alpha diversity indices were not different between the ham (corresponding to the top area in our study) and the jowl (bottom area). Despite bacterial count results that show a difference of the mesophilic aerobic bacteria between the top and bottom areas of the pig carcasses sampled during the current study, the beta diversity also seemed to be comparable between batches. This indicates that the differences in the intestinal microbiota reported in the literature between animals originating from different farms were not replicated on the carcass, suggesting that the primary processing, until the end of carcass dressing, globally standardized the pig carcass surface in terms of microbial diversity. This is important for pig farming as the modulation of gut microbiota to improve feed efficiency is being explored at the farm level. Based on our observations however, even with optimal primary processing practices, it seems that these attempts of gut microbiota modifications may not have any profound effects on carcass microbiota. This is also important for the control of bacterial carcass surface contamination at slaughterhouse as sources of contamination other than intestinal contents, such as processing line, slaughterhouse equipment or worker hands also contributes to the surface microbiota.

The results presented are specific to this study, which was conducted in a single slaughterhouse, on the same day, and at a single sampling point. This limits our ability to draw conclusions regarding the effect of farm origin in a universal manner. Differences may appear later in the processing, which deserves further attention. For example, during and after the cooling period, psychrotrophic bacteria are recognized to grow (Zwirzitz et al., 2020) and more differences may be observed beyond this point. The stability and impact of pig origin on final meat cuts should therefore also be investigated.

## Data Availability Statement

The datasets presented in this study can be found in online repositories. The names of the repository/repositories and accession number(s) can be found below: https://www.ncbi.nlm.nih.gov/, PRJNA757709.

## Author Contributions

CB: methodology, software, visualization, writing—original draft, and writing—review and editing. PF: conceptualization, supervision, writing—review and editing, and funding acquisition. M-LG: writing—reviewing and editing the manuscript. GL-G: resources, software, and writing—review and editing. FS-B: resources, software, and writing—review and editing. JL: resources, project administration, and writing—review and editing. AT: conceptualization, methodology, validation, supervision, project administration, resources, and writing—review and editing. All authors contributed to the article and approved the submitted version.

## Conflict of Interest

JL was employed by F. Ménard, Olymel LP. The remaining authors declare that the research was conducted in the absence of any commercial or financial relationships that could be construed as a potential conflict of interest.

## Publisher’s Note

All claims expressed in this article are solely those of the authors and do not necessarily represent those of their affiliated organizations, or those of the publisher, the editors and the reviewers. Any product that may be evaluated in this article, or claim that may be made by its manufacturer, is not guaranteed or endorsed by the publisher.

## Footnotes

1
https://mothur.org/wiki/miseq_sop/

2
https://www.mothur.org/wiki/Silva_reference_files

3
https://www.mothur.org/wiki/RDP_reference_files
