# Supplementary material for: Similar Carcass Surface Microbiota Observed Following Primary Processing of Different Pig Batches
Source: Front Microbiol. 2022 May 27;13:849883. doi: 10.3389/fmicb.2022.849883 (PMC9184759; doi:10.3389/fmicb.2022.849883)
Supplement: Supplementary file 1 [file Data_Sheet_1.docx]

Supplementary Material

**
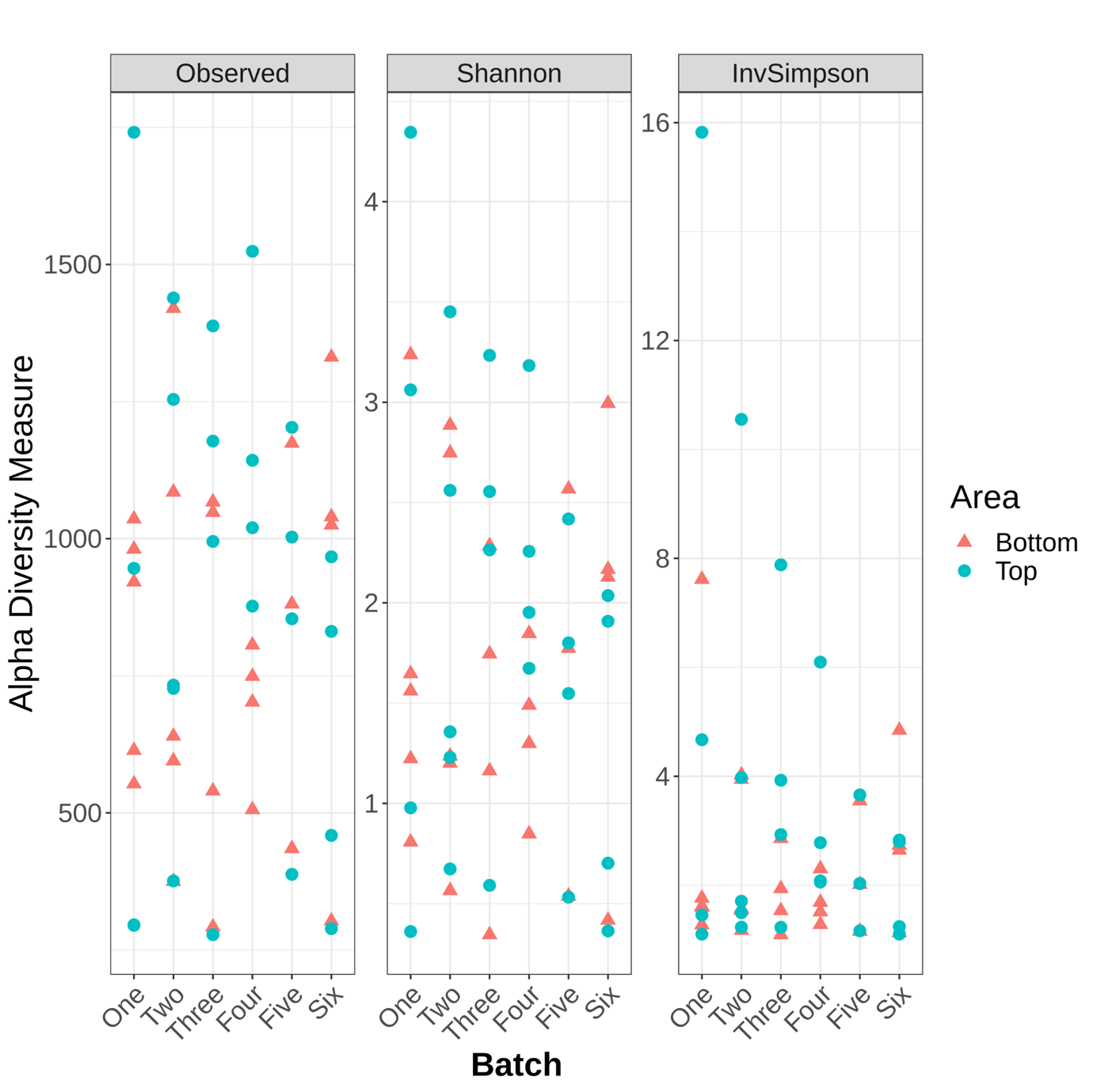
**

**Supplementary Figure 1.** Alpha diversity analyses using Observed, Shannon, and Inverse Simpson indices between the top and the bottom areas of pigs from six different batches.

**
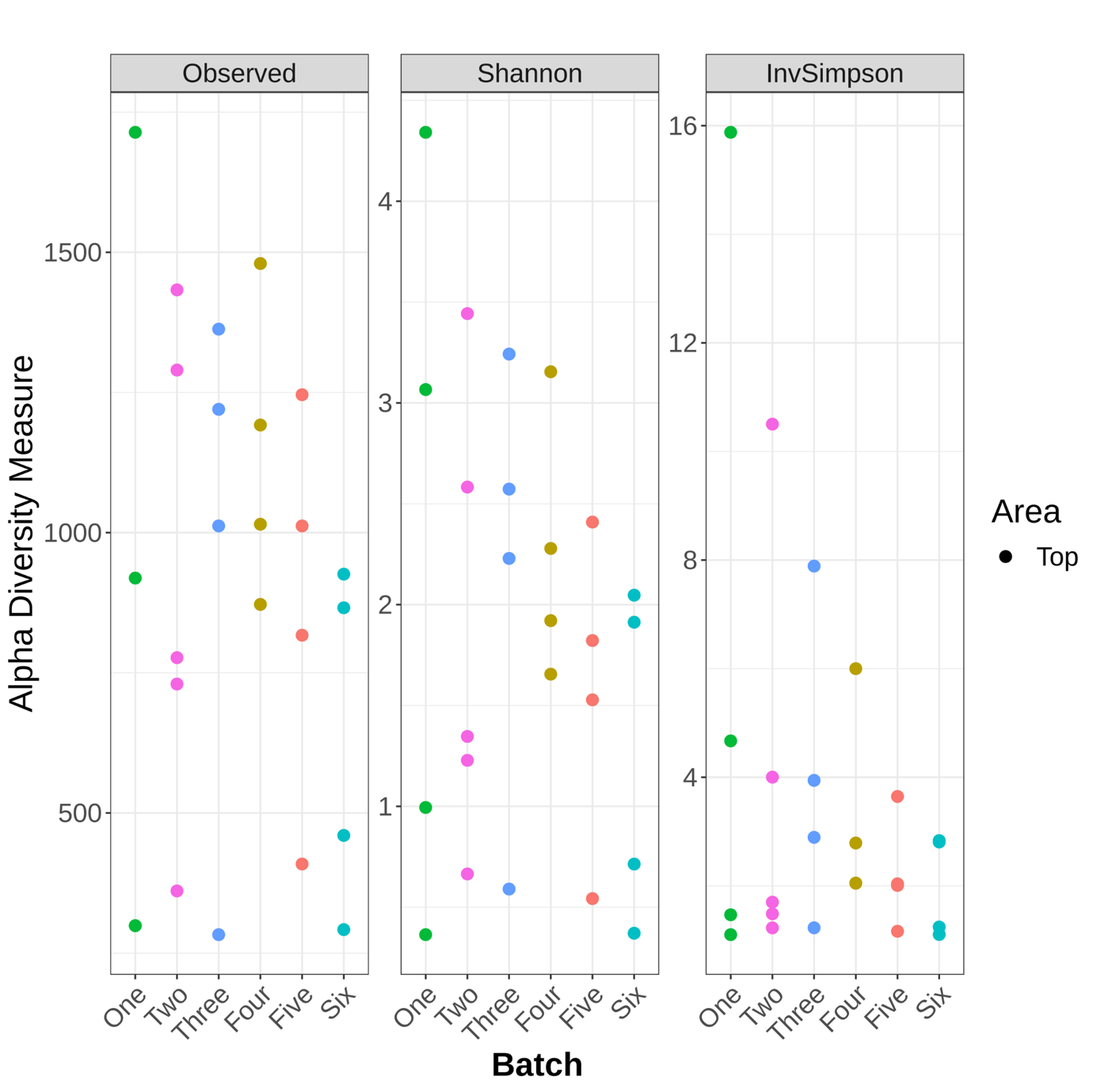
**

**Supplementary Figure 2.** Alpha diversity analyses using Observed, Shannon, and Inverse Simpson indices between the six batches and the top carcass surface samples.

**
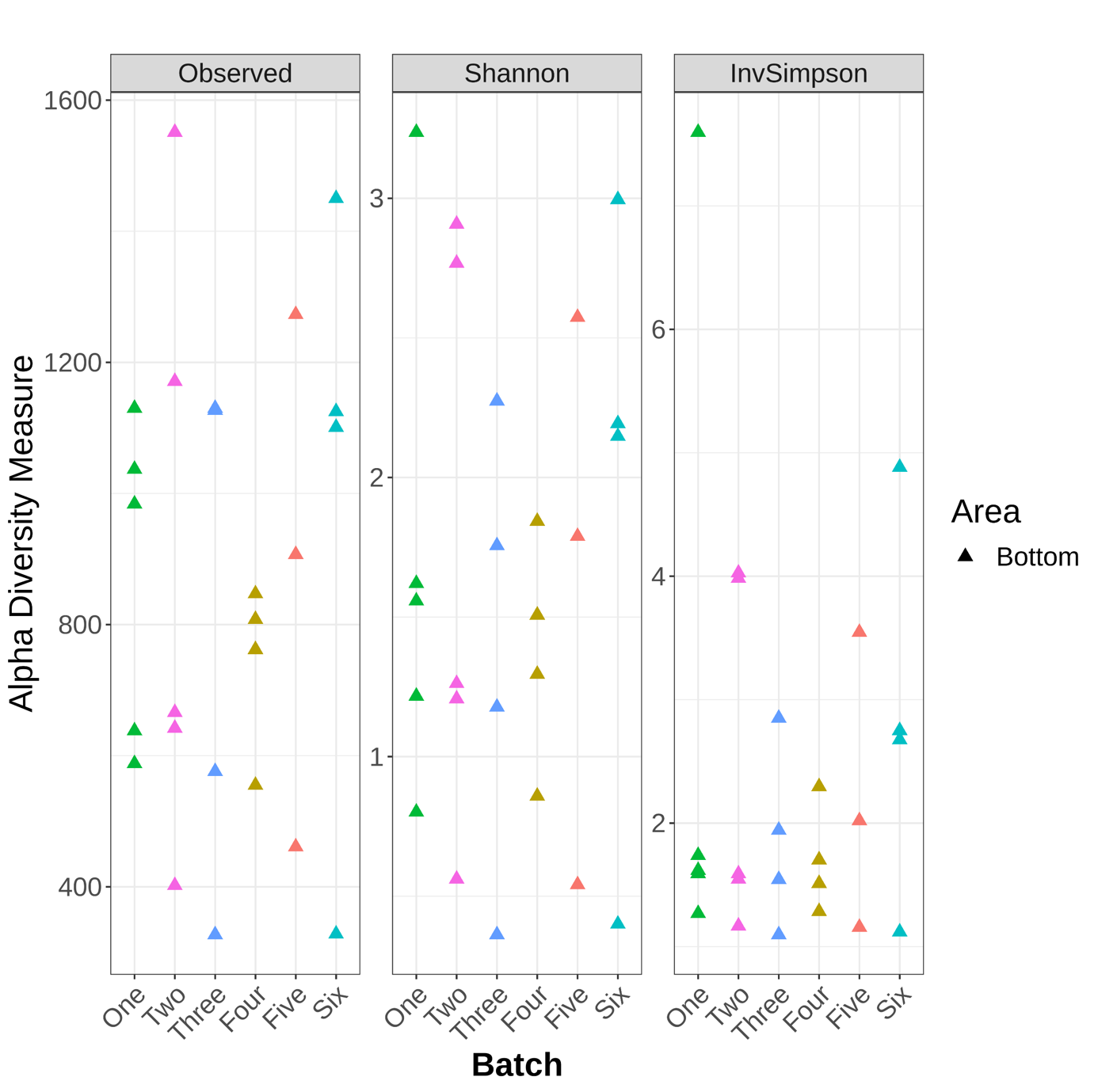
**

**Supplementary Figure 3.**Alpha diversity analyses using Observed, Shannon, and Inverse Simpson indices between the six batches and the bottom carcass surface samples.

**Supplementary Table 1.** Complete list of significant associations between carcass area (top or bottom) and relative microbial abundance using Maaslin2

| **Factor** | **Phylum** | **Family** | **Genus** | **Value** | **Coefficient** | **pval** | **qval** |
| --- | --- | --- | --- | --- | --- | --- | --- |
| Area | Proteobacteria | *Halomonadaceae* | *Halomonas* | Bottom | 8.91e-05 | 1.58e-08 | 9.36e-06 |
| Area | Proteobacteria | *Caulobacteraceae* | *Phenylobacterium* | Top | 0.00075 | 2.36e-06 | 0.00069 |
| Area | Proteobacteria | *Bradyrhizobiaceae* | *Bradyrhizobium* | Top | 0.0010 | 1.16e-05 | 0.0017 |
| Area | Firmicutes | *Streptococcaceae* | *Lactococcus* | Bottom | 0.00054 | 9.08e-06 | 0.0017 |
| Area | Firmicutes | *Aerococcaceae* | *Aerococcus* | Bottom | 0.00068 | 0.00015 | 0.015 |
| Area | Actinobacteria | *Corynebacteriaceae* | *Corynebacterium* | Bottom | 0.001 | 0.00014 | 0.015 |
| Area | Bacteroidetes | *Flavobacteriaceae* | *Cloacibacterium* | Top | 0.0003 | 0.0007 | 0.059 |
| Area | Proteobacteria | *Shewanellaceae* | *Shewanella* | Bottom | 3.21e-05 | 0.001 | 0.076 |
| Area | Proteobacteria | *Labilitrichaceae* | *Labilithrix* | Top | 4.89e-05 | 0.0015 | 0.09 |
| Area | Deinococcus-Thermus | *Deinococcaceae* | *Deinococcus* | Top | 0.0011 | 0.0019 | 0.10 |
| Area | Bacteroidetes | *Chitinophagaceae* | *Sediminibacterium* | Top | 3.50e-05 | 0.0027 | 0.12 |
| Area | Firmicutes | *Bacillales_Incertae_Sedis_XI* | *Gemella* | Bottom | 0.0001 | 0.0033 | 0.13 |
